# Optimizing the Equitable Deployment of Virtual Care for Women: Protocol for a Qualitative Evidence Synthesis Examining Patient and Provider Perspectives Supplemented with Primary Qualitative Data

**DOI:** 10.1089/heq.2023.0089

**Published:** 2023-09-13

**Authors:** Karen M. Goldstein, Dhara B. Patel, Katherine A. Van Loon, Abigail Shapiro, Sharron Rushton, Allison A. Lewinski, Tiera J. Lanford, Sarah Cantrell, Leah L. Zullig, Sarah M. Wilson, Megan Shepherd-Banigan, Susan Alton Dailey, Catherine Sims, Cheryl Robinson, Neetu Chawla, Hayden B. Bosworth, Alison Hamilton, Jennifer Naylor, Jennifer M. Gierisch

**Affiliations:** ^1^ VA Center of Innovation to Accelerate Discovery and Practice Transformation, Durham VA Healthcare System, Durham, North Carolina, USA.; ^2^ Division of General Internal Medicine, Department of Medicine, Duke University, Durham, North Carolina, USA.; ^3^ School of Nursing, Duke University, Durham, North Carolina, USA.; ^4^ School of Medicine, Duke University Medical Center Library, Durham, North Carolina, USA.; ^5^ Department of Population Health Sciences, School of Medicine, Duke University, Durham, North Carolina, USA.; ^6^ Department of Psychiatry and Behavioral Sciences, School of Medicine, Duke University, Durham, North Carolina, USA.; ^7^ Duke Margolis Center for Health Policy, Durham, North Carolina, USA.; ^8^ VA VISN-6 Mid-Atlantic Mental Illness Research and Education Clinical Center, Durham, North Carolina, USA.; ^9^ Department of Medicine, Division of Rheumatology, Duke University, Durham, North Carolina, USA.; ^10^ Clinical Translational Sciences Institute, School of Medicine, Duke University, Durham, North Carolina, USA.; ^11^ Veteran Research Engagement Panel, VA Center of Innovation to Accelerate Discovery and Practice Transformation, Durham, North Carolina, USA.; ^12^ VA Center for the Study of Healthcare Innovation Implementation and Policy, Los Angeles, California, USA.; ^13^ Department of Psychiatry and Biobehavioral Sciences, University of California Los Angeles David Geffen School of Medicine, Los Angeles, California, USA.

**Keywords:** virtual care, telehealth, women's health, antiracist, evidence synthesis, methods

## Abstract

**Introduction:**

Women experience numerous barriers to patient-centered health care (e.g., lack of continuity). Such barriers are amplified for women from marginalized communities. Virtual care may improve equitable access. We are conducting a partner-engaged, qualitative evidence synthesis (QES) of patients' and providers' experiences with virtual health care delivery for women.

**Methods:**

We use a best-fit framework approach informed by the Non-adoption, Abandonment, Scale-up, Spread, and Sustainability framework and Public Health Critical Race Praxis. We will supplement published literature with qualitative interviews with women from underrepresented communities and their health care providers. We will engage patients and other contributors through multiple participatory methods.

**Results:**

Our search identified 5525 articles published from 2010 to 2022. Sixty were eligible, of which 42 focused on women and 24 on provider experiences. Data abstraction and analysis are ongoing.

**Discussion:**

This work offers four key innovations to advance health equity: (1) conceptual foundation rooted in an antiracist action-oriented praxis; (2) worked example of centering QES on marginalized communities; (3) supplementing QES with primary qualitative information with populations historically marginalized in the health care system; and (4) participatory approaches that foster longitudinal partnered engagement.

**Health Equity Implications:**

Our approach to exploring virtual health care for women demonstrates an antiracist praxis to inform knowledge generation. In doing so, we aim to generate findings that can guide health care systems in the equitable deployment of comprehensive virtual care for women.

## Introduction

A solution is needed to address critical sex- and gender-based health disparities in the United States. Disparities disadvantaging women can be seen in health outcomes and health care utilization. For example, women have worse outcomes after acute coronary events compared with men.^1,2^ Compared with men, women have higher health care costs, are less likely to have access to a regular provider, and more likely to forgo needed care.^3,4^

Such disparities are amplified for women from historically marginalized communities such as women of color or women living in rural areas whose voices are often omitted from the design and optimization of health care systems and who often have worse health outcomes than white, urban-dwelling women. For example, in the United States, Black women experience a 3.5 times higher rate of maternal mortality compared with White women.^5^ Optimized delivery of evidence-based sex-specific, gender-informed health care could reduce existing disparities if designed to overcome logistical barriers, reduce discrimination, and promote the equitable provision of health care.^6,7^

One way to deliver such care would be to leverage virtual modalities to connect women with knowledgeable, patient-centered health care teams in the right way, at the right time.^3,8^

Optimizing virtual care for women requires an understanding of how women with different identities and life experiences engage with, and experience, virtual health care delivery. First and foremost, an individual's gender (a social construct encompassing gender identity and expression along with social and cultural expectations about role, power, status, characteristics, and behaviors) may impact a woman's experience of virtual care separately from sex (biological identity assigned at birth).^9^ Furthermore, an understanding of women's preferences for use of virtual care for both sex-specific (e.g., pregnancy) and sex-neutral conditions (e.g., cardiovascular disease) is critical.

Beyond sex and gender, any individual woman faces additional intersecting systems of marginalization based on other identities and life experiences. For example, evidence from the early COVID-19 pandemic suggests women were more likely to use virtual visits than men regardless of race {Cherabuddi, 2023 #10}; this could create opportunities to improve the care provided to women of color in some important types of emerging digital care such as prenatal telehealth, which have the potential to address critical disparities in women's health outcomes.

How information is shared is an important determinant of peripartum health care experiences for women of color {Altman, 2019 #11}, thus a purposeful, intersectional lens to the structuring of digital health systems is crucial to creating telehealth experiences that can overcome longstanding patterns of discrimination in women's health care {Figueroa, 2021 #12}. In addition to racial or ethnic disparities, individuals living in rural areas are less likely to have adequate broadband access to support participation in virtual care {Shreck, 2020 #13}, highlighting how social determinants (e.g., rurality, housing, socioeconomic resources, etc.) can key intersect with social identities to impact equitable access to optimized virtual care for women. For example, in a study of Black, rural-dwelling Veterans, video-based care was believed to be of lower quality than in-person care, and offering virtual care was perceived as the provision of substandard health care.^14^ Thus, indiscriminate implementation of virtual care for racially diverse populations of women has the potential to alienate women who may already have mistrust of the health care system rooted in historical mistreatment due to structural racism.

Furthermore, many women with histories of trauma experience barriers to meaningful engagement with health care providers and services. Thus, trauma-sensitive communication strategies and informed-care principles are needed to optimize care delivery for women patients with trauma histories.^15–17^

Virtual care experiences are also driven by the health care system in which it is delivered. The Department of Veterans Affairs (VA) offers an ideal setting in which to consider the optimal delivery of virtual care for women due to its robust, long-standing telehealth infrastructure,^18^ and an intentional, comprehensive approach to women's health care delivery.^19,20^ While the implementation of the VA's approach to women's health care has improved care quality, logistical barriers to sex-specific access to care persist.^21,22^

Virtually delivered care is a possible solution to overcome access barriers for women veterans.^8,22^ In fact, during the COVID-19 pandemic, women veterans were more likely to become new video visit users than male veterans.^23^ Yet, the population of women Veterans cared for in the VA is racially diverse, has a greater proportion of Lesbian, Gay, Bisexual, Transgender, Queer or Questioning, Intersex, Asexual and more (LGBTQIA+) identifying individuals than civilian practices, and has a high prevalence of comorbid health concerns (e.g., prior trauma history, comorbid mental health conditions),^24,25^ factors which are known to impact engagement with health care.^26^

We seek to explore the diversity of experiences of either receiving or delivering virtual health care for women using a theory-driven intersectional and antiracist approach. Thus, we will synthesize the perspectives of women recipients of virtual care and those of health care team members (e.g., clinicians, schedulers, administrators). The key question driving this work is: *What are the successes and challenges to accessing, delivering, and engaging in synchronous virtual health care for women?*

## Methods

**FIG. 1.**
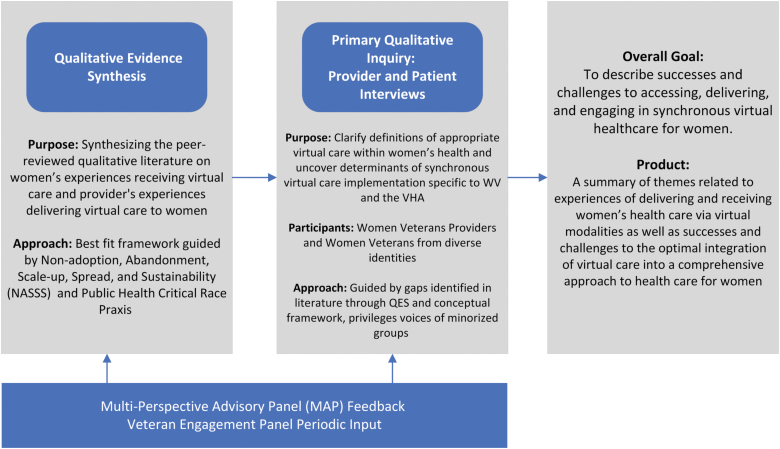
Methods overview for understanding the experience of receiving and delivering synchronous virtual care to women. QES, qualitative evidence synthesis; VHA, Veterans Health Administration; WV, Women Veterans.

Given the maturity of the existing literature and nature of our question, we will first conduct a qualitative evidence synthesis (QES) of peer-reviewed qualitative literature about women's experiences with virtually delivered health care and those of health care team members who deliver that care (Fig. 1). We anticipate that perspectives available in the published literature will not represent the full diversity of women patients' identities, particularly related to differential access to high-quality care. Thus, we plan to supplement the QES during a second phase in which we collect primary qualitative data using semistructured interviews with individuals not well represented in the literature and from minoritized populations. Specifically, we will seek to include women veterans, women from historically marginalized racial/ethnic identities, individuals who were not assigned female at birth who identify as women and rural-dwelling women, and VA health care team members who have some role in virtual health care delivery.

Throughout, we define virtual care as synchronous health care delivered through telephone or videoconferencing that involves bidirectional information exchange between a patient and a health care provider (i.e., physician, nurse). We will follow enhancing transparency in reporting the synthesis of qualitative research reporting guidelines for the QES^27^ and have registered an *a priori* protocol with PROSPERO (CRD42021283791).

The work described here will serve as foundational knowledge to inform the development of an implementation blueprint to optimized equitable delivery of synchronous virtual care for women veterans. Women remain an important, understudied, and growing population within the VA patient community.

### Partnered engagement

Multiple opportunities exist to engage diverse partners in the conduct of evidence synthesis.^28^ We will enrich this work with partnered engagement through three pathways. First, we benefit from a core study team that includes women of color, VA health care providers, and women patients. Second, we have received, and will continue to seek, input from a patient engagement panel at our institution. This group comprises diverse patient partners, of which nearly 50% are women veterans. Finally, we have developed a study-specific multiperspective advisory panel (MAP) through which we will receive semiannual input from the lived and learned expertise of women veterans of color from diverse branches and eras of service, and health care professionals representing various roles in the VA health care system.

### Conceptual framework

**FIG. 2.**
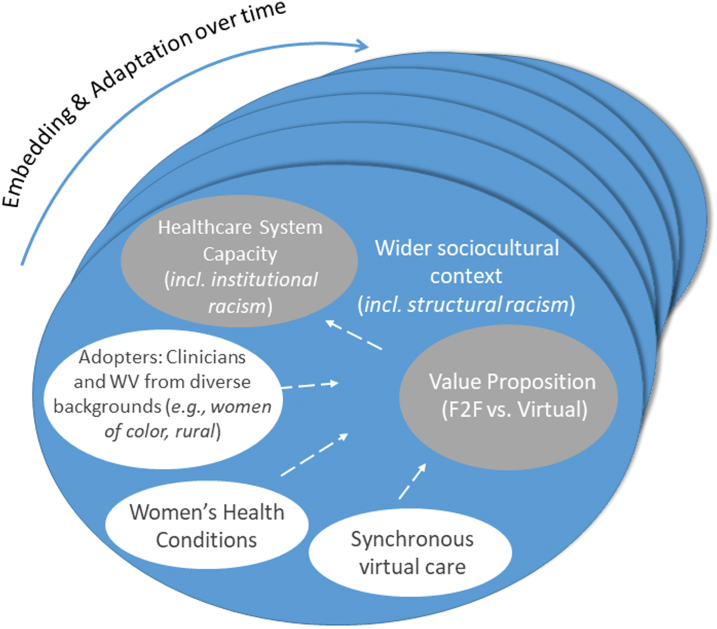
Conceptual framework: adapted Nonadoption, Abandonment, Scale-up, Spread, and Sustainability. Reproduced with permission from Greenhalgh et al.^25^ F2F, face to face; Incl., including.

Our conceptual foundation is rooted in a dynamic antiracist and action-oriented praxis to inform the equitable deployment of virtual care for women. First, as our focus is on optimal implementation of virtual care, we will use the nonadoption, abandonment, scale-up, spread, and sustainability (NASSS) technology implementation framework.^29^ The NASSS is an evidence-based, theory-informed framework built to provide practical implementation guidance for technology-supported health innovations. NASSS encompasses six critical domains: (1) health condition, (2) technology, (3) value proposition, (4) adopter system, (5) organization, and (6) wider (institutional and societal) context. NASSS also includes a seventh domain that accounts for interactions between domains and adaptations over time. The NASSS domains will provide structural codes for successes and challenges to virtual care during the QES and inform interview guides for our primary qualitative work with women veterans and VA health care team members (Fig. 2).

We also include an intentional focus on equity to ensure that we do not disproportionately benefit women from privileged groups or perpetuate structural bias in health care system design through our findings.^30^ As such, our work is also informed by Public Health Critical Race Praxis (PHCRP).^31^ The PHCRP is an applied extension of Critical Race Theory,^32^ and is an iterative methodology for facilitating action-oriented research with an antiracist and equity lens. Public Health Critical Race (PHCR) focuses on race as a social construct and conceptualizes race as a marker for racism-related exposures (e.g., discrimination) rather than an individual demographic characteristic. Our work is informed by the theory's principles of race consciousness, intersectionality, social construction of race, primacy of racialization, ordinariness of racism, and voice. Table 1 illustrates examples of how PHCRP informs this study. This methodology aligns with recommended approaches for QES to seek a diversity of data sources to reduce bias from focusing on the dominant perspective most commonly identified in the literature.^33^

**Table 1.** Examples of Public Health Critical Race Praxis Integration in Research Approach

PHCRP principle and definition^31,^^50^	Example in research approach
Race consciousness: awareness of one's racial position	• Over the course of the study, researchers will interrogate our own racial biases and salience of colorblind concepts • Assess included studies in the QES for statements of positionality • Racially diverse research team • When feasible, conducting qualitative interviews with racially concordant research staff
Intersectionality: interactive nature of co-occurring social categories (e.g., gender, race)	• Intentional sampling of studies for the QES focused on studies with intersectional populations • Seeking diverse women for the MAP with intersectional identities • Exploring intersectional identities in primary qualitative interviews
Race as a social construct: significance of race as constructed by social, historical, and political factors Primacy of racialization: fundamental contribution of racial stratification to societal problems Ordinariness of racism: racism as normal and not uncommon, rather than an overt or rare events	• Used to build structural codes for QES and primary qualitative data to explore factors related to structural racism, and race, and other marginalized identities, as a marker for exposure to racism and discrimination • Analysis and interpretation assume racism and discrimination as part of the normal, yet influential, barrier of women seeking health care
Voice: prioritizing the perspectives of marginalized groups and privileging experiential knowledge	• Use of qualitative methods that privilege experiential knowledge of women and frontline health care members • Adding a primary qualitative approach focused on women of color to complement QES findings • Seeking guidance on the conduct of the study from women of color through engagement of the MAP, woman of color coinvestigator, and patient engagement panel. • Prioritizing articles for inclusion in the QES if centered on perspectives from underrepresented groups

MAP, multiperspective advisory panel; PHCRP, Public Health Critical Race Praxis; QES, qualitative evidence synthesis.

Thus, this work seeks to explore the way in which structural racism is expressed in the virtual care environment and will create opportunity for identifying clinical settings in which racial bias impedes high-quality virtual care for women.

### Qualitative evidence synthesis

#### Information sources and search strategy methods

MEDLINE (through Ovid), Embase (through Elsevier), and CINAHL Complete (through EBSCO) were searched from January 1, 2010 to October 10, 2022 using a combination of database-specific controlled vocabulary terms and keywords related to virtual care and women or aspects of traditionally gendered care. An experienced medical librarian (S.C.) devised and conducted the searches, with input from the other authors. The search used a modified qualitative research filter, adapted from the ASTED(3S)/CHLA.^34^ The search strategies were peer-reviewed by another librarian before the execution using a modified Peer Review of Electronic Search Strategies checklist.^35^ The full, reproducible search strategies are in Appendix A section of Supplementary Data. Additional references were identified by handsearching the VA women's health and telehealth listservs for relevant recent articles. All citations were imported into Covidence systematic review software (Veritas Health Innovation, Melbourne, Australia).

#### Eligibility criteria and screening

We used Sample, Phenomenon of Interest, Design, Evaluation, Research Type to structure eligibility criteria (Appendix B section in Supplementary Data). Our eligibility criteria focus on studies of synchronous qualitative data collection around the delivery of virtual care for women conducted in countries that resemble the United States with respect to economic criteria and health care resources. Included articles address bidirectional information exchange between a provider or clinical team and a patient for individualized care. We excluded studies featuring asynchronous virtual interactions (e.g., mHealth portals), men only, or mixed-gender studies where data were not analyzed by gender.

We used Covidence as a tool to screen articles and EndNote to organize our citations (Endnote, Clarivate Analytics, Philadelphia, PA). All articles were screened at the title and abstract and full-text levels by two independent investigators. Conflicts were resolved through discussion or arbitration by a third reviewer. We piloted each level of screening to ensure concordance across team members. At full-text review, articles were tagged based on their health care delivery pathway (e.g., mental health) and for focus on a marginalized population (e.g., rural, people of color, LGBTQIA+, veterans). These tags identified collections from which we sampled studies for abstraction. To identify those articles likely to provide richer qualitative data, qualitative analysts (A.S., T.J.L.) rated included articles on a richness of data scale (range: 1–5).^36^

#### Data abstraction

The data will be abstracted in two phases: first, abstraction of study characteristics in Covidence and, second, abstraction of qualitative findings in NVivo 12 (QSR International, released March 2020). A single abstractor with independent over-reading for data verification will be used. Key study characteristics include demographics, clinical setting, theoretical framework, and findings. Discrepancies will be resolved through consensus or by consulting a third team member. In keeping with standard QES methods, we will select a subset of articles for abstraction from all that pass full-text review stage.^36^ To ensure a diversity of information, we will sample studies from each of the core women's health care delivery pathways (i.e., primary care, Obstetrics and Gynecology (OB-GYN), non-OBGYN specialty, and mental health). These sampling strata were identified through internal research team discussion, by eliciting MAP input, and from internal team knowledge of care delivery for women.

We will sample approximately eight articles from each care pathway, prioritizing articles that include *a priori* marginalized population and articles with higher richness scores. We will continue to sample from the care pathway strata until we have sufficient saturation of findings (i.e., no new findings emerging from the data).

#### Data synthesis

The research team, led by two qualitative analysts, will develop an *a priori* codebook using the NASSS framework and the PHCRP as the foundation for a “best fit” framework synthesis approach.^37^ The codebook will contain relevant domains and constructs, definitions, and examples. Six coders will independently pilot code an initial two articles and meet to resolve conflicts with discussion and consensus. The team will code one additional article to test intercoder reliability, which will be judged on conceptual similarity rather than a Kappa statistic-based approach, which has serious limitations for use in qualitative research.^38–40^ Then, a subset of the codebook will be assigned to each coder, who will review all remaining articles for those codes. The team will meet regularly to discuss progress and challenges; disagreements will be resolved by consensus. An additional 10% of articles will be reviewed for quality assurance across domains.

#### Quality assessment

We will assess quality of included articles using the Critical Appraisal Skills Program by two independent reviewers; conflicts will be resolved by consensus.^41^ This tool considers these broad areas: appropriateness of methods (e.g., clarity of aims, appropriate design), presentation, and meaningfulness of findings (e.g., clarity of findings).^42^

#### Certainty of evidence

We will determine the level of confidence for our findings using the Confidence in the Evidence from Reviews of Qualitative Research (CERQual) approach.^43^ This approach considers the methodologic limitations of included studies, relevance of included studies, coherence of the review finding, and adequacy of the identified data with respect to the finding. We will work with our MAP to prioritize the five most crucial constructs in decision making for the implementation of synchronous virtual care for women and then apply CERQual to those prioritized constructs.

### Primary qualitative data collection

We plan to collect primary qualitative data to supplement our QES because, we anticipate that most literature identified in the QES will come from populations of White women. Including diverse voices is critical to the external validity of our work because women veterans have greater race/ethnicity and gender diversity than either civilians or male veterans.^24^ Thus, we will center our primary qualitative inquiry on underrepresented women using a maximum variation sampling plan to obtain diverse perspectives on the determinants of virtual care uptake. This approach is in keeping with recommended approaches for QES to seek a diversity of data sources to reduce bias from overly focusing on the dominant perspective most commonly identified in the literature.^33^ We will recruit women patients from the Durham and Greater Los Angeles VA Health Care Systems, where our team members are located. We plan to conduct ∼36 semistructured qualitative interviews: 24 with women patients from populations underrepresented in the QES and 12 with health care team members. The sampling strategy will be based on emerging gaps in perspectives from the QES and guidance from our MAP.

Interview guides will be informed by our conceptual foundation, as described above, and the findings from our QES (see Appendix C section in Supplementary Data for our preliminary interview guide).

#### Primary qualitative data analysis

We will employ an exploratory sequential approach to our primary qualitative work stemming from the findings of the QES.^44^ We will analyze the primary qualitative data using a rapid analytic approach.^45,46^ Summary templates will be reviewed weekly to bolster confirmability, trustworthiness, dependability, and credibility. Summary information will be transferred to matrices organized by informant and interview domains to facilitate data condensation and synthesis, paying attention to similarities, differences, and trends in responses across informant type.^47^ Initially, matrices will be developed for each informant category (i.e., patient, provider). For patient interviews, we will further stratify and analyze by self-identified racial/ethnic group. We will ensure the validity and reliability of findings and the iterative generation of codes through regular team discussions. We will systematically capture team reflections in templated analytic memos^46^ and code the matrix using codes drawn from NASSS and PHCR through independent coders. Investigators will maintain an audit trail of coding and analytic decisions.

Lastly, we will integrate the findings of the primary qualitative research with the findings with the QES, building on the results of the QES to develop a more robust and equitable understanding of the state of virtual care for women.

### Positionality statement

Our approach was largely informed by the clinical and research experiences of the first and senior authors who noticed a lack of equitable and optimized virtual care for women. The lead and senior authors are cisgender, white women who hold terminal degrees and have family who served in the military. There are five women of color authors on this article, including one patient coresearcher. Several women of color serve on the MAP.

## Results

**FIG. 3.**
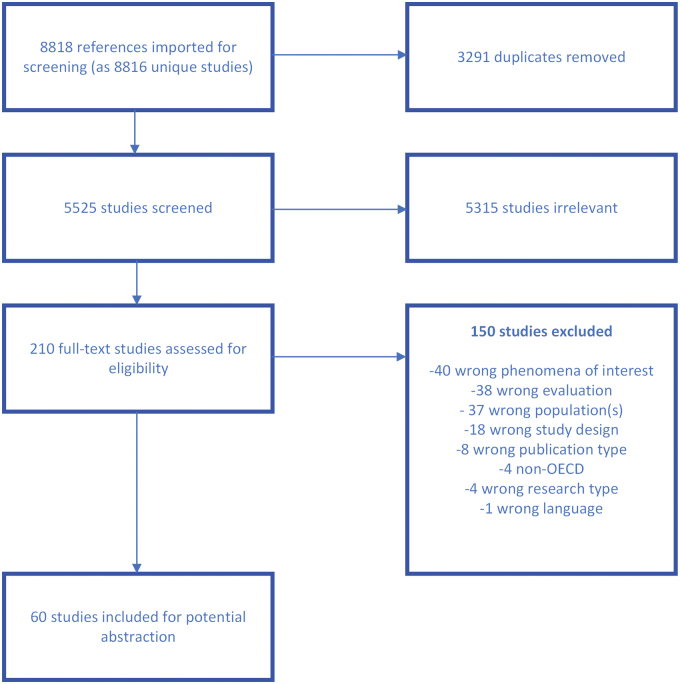
QES literature flow. Non-OECD, Non-Organization for Economic Co-operation and Development.

**FIG. 4.**
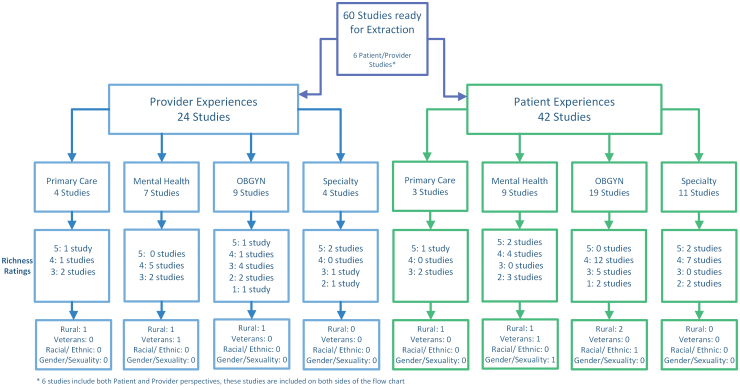
QES sampling structure for included articles.

We identified 8818 studies through our search (Fig. 3) After removing duplicates there were 5525 studies, of which 210 studies were reviewed at full text. Sixty studies were retained for abstraction, of which 24 are focused on provider experiences, 42 on patient experiences, and 6 studies included both perspectives. Following categorization by provider and patient experiences, the articles were stratified by the type of care provided to prepare for sampling (Fig. 4). Of the 60 included articles, 3 explicitly focused on a marginalized population, with 2 focused on racial/ethnic underrepresented groups, and 1 on gender/sexuality minorities. The proportion of women from racial/ethnic minoritized populations ranged from 0% to 100%, with the majority (67%) of articles falling below 50%. Fifteen articles did not report race/ethnicity of participants. Two articles focused on women veterans and six on rural-dwelling women. Data abstraction is currently ongoing.

## Health Equity Implications and Discussion

Our methodologic approach offers four key scientific contributions at the intersection of health equity and evidence synthesis methods.

First, we demonstrate an innovative antiracist and antibiased praxis to explore virtual health care for women. The rapid uptake in virtual care during the COVID-19 pandemic exposed widespread disparities in access to virtual care, largely among older adults and those living in rural and lower income communities.^10,48^ Our methods and team processes, are guided by an internal focus of emphasizing perspectives of socially marginalized groups rather than the perspectives of the dominate cultural group. Such an approach highlights opportunities to reduce disparities by identifying ways to improve access and building clinic-based virtual practices that enhance equitable and trustworthy care. For example, if women of color report that receiving initial provider visits in a virtual care environment hinders the ability to develop a trusting therapeutic relationship, this might suggest the need for a standardized workflow to ensure appropriate scheduling of visit modality for new patients to mitigate implicit bias in the way that virtual visits are allocated.

Second, we offer a worked example of centering a QES on marginalized communities. The purpose of a QES is to summarize the existing primary qualitative literature about a specific phenomenon, or set of experiences or circumstances, to develop a more subtle understanding than allowed by individual primary qualitative study.^49^ In this study, we seek to promote a subtle understanding of women's experiences of virtual care centered on minoritized communities. The focus on minoritized communities is particularly important for women veterans who have greater diversity of intersectional racial and ethnic identity and sexual orientation, as well as rural-dwelling status, than the general U.S. population.^24^ We will accomplish this by using deliberate methodologic approaches across our search terms, sampling approach, coding, and analysis framework that specifically prioritize populations typically left at the margins of health care innovation. Furthermore, we integrate antiracist team processes informed by PHCRP.

Third, we will supplement QES with strategic primary qualitative data collection to ensure inclusion of populations underrepresented in the published literature. Previous researchers have sought systematic review strategies to overcome inherent biases of QES methodologies. Yet, due to academic traditions that tend to dominate peer-reviewed literature (e.g., Western dominance), these approaches were not able to yield a diversity of perspectives.^33^ Through supplementing QES with primary qualitative data collection from select underrepresented populations, we are further grounding our findings in voices that would likely be missed if our efforts were limited to peer-reviewed literature.

Lastly, we fortify our approach and analysis through longitudinal participatory and partnered engagement at multiple points. For a patient perspective, we benefit from a patient coresearcher on our core study team, intersectionally diverse patient members of the MAP, and will continue to obtain iterative feedback from an established veteran engagement panel at our institution. For health care team member insights and input, we engage with the study-specific MAP comprising key partners and leaders from relevant administrative offices within the VA, and clinical study team members who care for women Veterans.

Our approach is limited by its tailoring to the VA health care system context. In this way, it will be most relevant to other large, comprehensive health care systems. Our methods may be less applicable to smaller, fee-based health care systems or systems that are not as diverse as the VA. Yet, we have elected to focus our approach to shorten translation from scientific product to direct impact in the VA environment. Also, we are only including articles with data collected from English-speaking individuals, so our findings may not apply to women with language-based barriers. Finally, our research team does not include trans or nonbinary members. We acknowledge our inability to understand trans and nonbinary experiences from a lived experience standpoint and seek to uplift gender-diverse experiences through our QES and primary qualitative data inquiry.

## Conclusions

Virtual care modalities offer an opportunity to improve access to sex-specific and gender-informed women's health in a manner that is convenient and equitable. An important initial step to the equitable deployment of virtual care is understanding the experiences of women and their health care teams around synchronous care delivery through technology-driven modalities. Ensuring these experiences reflect the intersectional diversity of women who can benefit from virtual care will provide key information to inform its equitable provision. To achieve this, we describe an innovative approach to knowledge generation through incorporation of a theory-driven, antiracist approach, centering our data collection on the margins, supplementing the published literature with strategic data collection from underrepresented voices, and grounding with longitudinal partnered engagement.

## Supplementary Material

Supplemental data

## Notes

**Cite this article as:** Goldstein KM, Patel DB, Van Loon KA, Shapiro A, Rushton S, Lewinski AA, Lanford TJ, Cantrell S, Zullig LL, Wilson SM, Shepherd-Banigan M, Alton Dailey S, Sims C, Robinson C, Chawla N, Bosworth HB, Hamilton A, Naylor J, Gierisch JM (2023) Optimizing the equitable deployment of virtual care for women: protocol for a qualitative evidence synthesis examining patient and provider perspectives supplemented with primary qualitative data, *Health Equity* 7:1, 570–580, DOI: 10.1089/heq.2023.0089.

## Abbreviations Used

CERQual: Confidence in the Evidence from Reviews of Qualitative Research
F2F: face to face
HSR&D: Health Services Research and Development
Incl.: including
MAP: multiperspective advisory panel
NASSS: nonadoption, abandonment, scale-up, spread, and sustainability
Non-OECD: Non-Organization for Economic Co-operation and Development
PHCR: Public Health Critical Race
PHCRP: Public Health Critical Race Praxis
QES: qualitative evidence synthesis
VA: Department of Veterans Affairs
VHA: Veterans Health Administration
WV: Women Veterans
