# Supplementary material for: Optimizing the Equitable Deployment of Virtual Care for Women: Protocol for a Qualitative Evidence Synthesis Examining Patient and Provider Perspectives Supplemented with Primary Qualitative Data
Source: Health Equity. 2023 Sep 13;7(1):570–80. doi: 10.1089/heq.2023.0089 (PMC10507937; doi:10.1089/heq.2023.0089)
Supplement: Supplemental data [file Suppl_Data.docx]

**Appendix A. Search Strategy**

HEROES project

Librarian searcher: Sarah Cantrell, MLIS; Duke University Medical Center Library & Archives; Duke University School of Medicine

Peer-review conducted by: Brandi Tuttle, MSLS; Duke University Medical Center Library & Archives; Duke University School of Medicine

**Database: MEDLINE (via Ovid)**
Search date: 10/10/2022
*Note: Ovid MEDLINE(R) ALL 1946 to October 07, 2022*

| **Description** | **Search Set** | **Search Strategy** | **Results** |
| --- | --- | --- | --- |
| *Virtual Care terms* | #1 | exp Telemedicine/ or exp Remote Consultation/ or Videoconferencing/ or Telephone/ or Cell phone/ or Smartphone/ | 69,960 |
|  | #2 | (telehealth or tele-health or telemedicine or tele-medicine or telemedical or tele-medical or telecare or tele-care or teleprimary care or tele-primary care or tele-PCP or tele-visit or televisit or tele-visits or televisits or teleconference or tele-conference or teleconferences or tele-conferences or telemanage or tele-manage or telemanagement or tele-management or telepharmacy or tele-pharmacy or telepharmacies or tele-pharmacies or telepharmacist or telepharmacists or tele-pharmacist or tele-pharmacists or telegynecology or tele-gynecology or telegynecologist or tele-gynecologist or telegynecologists or tele-gynecologists or teleobstetrics or tele-obstetrics or teleobstetrician or tele-obstetrician or teleobstetricians or tele-obstetricianstelenurse or tele-nurse or tele-nurses or tele-nursing or telenurse or telenurses or telenursing or telepsychiatrist or tele-psychiatrist or telepsychiatrists or tele-psychiatrists or telepsychiatry or tele-psychiatry or telecounselling or tele-counselling or telecounselor or tele-counselor or teleconselors or tele-counselors or teleintervention or tele-interventions or teleinterventions or tele-interventions or tele-therapy or tele-therapies or teletherapy or teletherapies).ti,ab. | 29,047 |
|  | #3 | ((virtual or virtually or video or video-based or videobased or videoconference or video-conference or videoconferences or video-conferences or videoconferencing or video-conferencing or webconference or web-conference or webconferences or web-conferences or webconferencing or web-conferencing or Zoom or Skype or WebEx or FaceTime or GoToMeeting or "web based" or web-based or webbased or online or telephone or tele-phone or telephones or tele-phones or telephoned or tele-phoned or cellphone or cellphones or cell-phone or cell-phones or "cell phone" or "cell phones" or smartphone or smartphones or smart-phone or smart-phones or "smart phone" or "smart phones" or "cellular phone" or "cellular phones" or "mobile device" or "mobile devices" or "mobile phone" or "mobile phones" or iPhone or iPhones or iPad or iPads or Android) adj5 (care or cared or health or healthcare or appointment or appointments or meeting or meetings or met or meet or visit or visits or clinic or clinics or medicine or medical or therapy or therapies or therapeutic or therapeutics or intervention or interventions or treatment or treatments or treat or treats or treated or manage or manages or management or managed or physician or physicians or clinician or clinicians or doctor or doctors or patient or patients or nurse or nurses or nursing or diagnose or diagnosis or diagnoses or diagnostic or prescribe or prescribes or prescribing or prescription or prescriptions or pharmacy or pharmacies or pharmacist or pharmacists or counsel or counsels or counselled or counselling or counsellor or counsellors or psychiatry or psychiatrist or psychiatrists or "mental health" or gynecology or gynecologist or gynecologists or obstetrics or obstetrician or obstetricians or "OB/GYN" or OBGYN or "OB GYN")).ti,ab. | 97,668 |
|  | #4 | (Tele adj2 (care or cared or health or healthcare or appointment or appointments or meeting or meetings or visit or visits or clinic or clinics or medicine or medical or session or sessions or therapy or therapies or therapeutic or therapeutics or intervention or interventions or treatment or treatments or treat or treats or treated or manage or manages or management or managed or physician or physicians or clinician or clinicians or doctor or doctors or nurse or nurses or nursing or diagnose or diagnosis or diagnoses or diagnostic or prescribe or prescribes or prescribed or prescribing or prescription or prescriptions or pharmacy or pharmacies or pharmacist or pharmacists or psychiatry or psychiatrists or psychiatrist or "mental health" or counselling or counsel or counsels or counselled or counsellor or counsellors or gynecology or gynecologist or gynecologists or obstetrics or obstetrician or obstetricians or "OB/GYN" or OBGYN or "OB GYN")).ti,ab. | 1,271 |
|  | #5 | ((remote or remotely) adj3 (care or cared or health or healthcare or appointment or appointments or meeting or meetings or visit or visits or therapy or therapies or therapeutic or therapeutics or intervention or interventions or treatment or treatments or treat or treats or treated or management or managed or diagnose or diagnosis or diagnoses or diagnostic or prescribe or prescribes or prescribed or prescribing or prescription or prescriptions or "mental health" or counselling or counsels or counselled or counsel or gynecology or gynecologist or gynecologists or obstetrics or obstetrician or obstetricians or "OB/GYN" or OBGYN or "OB GYN")).ti,ab. | 7,487 |
| *combining* | #6 | 1 or 2 or 3 or 4 or 5 | 167,204 |
| *Women or women's health terms* | #7 | exp Women/ or exp Women's Health/ or exp Women's Health Services/ or exp Health Services for Transgender Persons/ or exp Homosexuality, Female/ or (woman or women or womens or womans or "women s" or "woman s" or Female or females or "female s" or transwoman or trans-woman or transwomans or "transwoman s" or trans-womans or "trans-woman s" or trans-women or transwomen or transwomens or "transwomen s" or trans-womens or "transwomen s" or non-binary or nonbinary or transman or trans-man or transmans or "transman s" or trans-mans or "trans-man s" or transmen or trans-men or transmens or trans-mens or "transmen s" or "transmen s" or trans or transgender or transgendered or lesbian or lesbians).ti,ab. | 2,468,379 |
|  | #8 | exp Breast Diseases/ or exp Breast Neoplasms/ or ("breast health" or "breast disease" or "breast diseases" or "breast cancer" or "breast cancers" or "breast neoplasm" or "breast neoplasms").ti,ab. | 441,152 |
|  | #9 | exp Pregnant Women/ or exp Maternal Health/ or exp Maternal Health Services/ or exp Prenatal Care/ or exp Perinatal Care/ or exp Postnatal Care/ or exp Postpartum Period/ or exp Depression, Postpartum/ or exp Pregnancy/ or exp Pregnancy Complications/ or exp Breast Feeding/ or exp Lactation/ or (pregnancy or pregnancies or pregnant or pregnancy-induced or pregnancy-associated or prenatal or pre-natal or prenatally or pre-natally or perinatal or peri-natal or perinatally or peri-natally or postnatal or post-natal or postnatally or post-natally or maternal or maternally or postpartum or post-partum or breastfeeding or "breast feeding" or lactation).ti,ab. | 1,413,236 |
|  | #10 | exp Contraception/ or exp Hormonal Contraception/ or exp Contraceptives, Oral/ or exp "Contraceptive Devices, Female"/ or Reproductive Health Services/ or exp Preconception Care/ or exp Family Planning Services/ or exp Fertility/ or exp Infertility/ or exp Fertility Clinics/ or exp Abortion, Induced/ or exp Abortion, Spontaneous/ or exp Levonorgestrel/ or (conception or preconception or pre-conception or "reproductive health" or "reproductive care" or "reproductive healthcare" or "reproductive plan" or "reproductive planning" or "family planning" or fertility or infertility or contraception or contraceptive or contraceptives or "morning after pill" or "morning after pills" or levonorgestrel or "plan b" or abortifacient or abortifacients or misoprostol or mifepristone or RU-486 or abortion or abortions).ti,ab. | 422,363 |
|  | #11 | exp Menopause/ or (menopause or menopausal or perimenopause or peri-menopause or perimenopausal or peri-menopausal or premenopause or pre-menopause or premenopausal or pre-menopausal or postmenopause or post-menopause or postmenopausal or post-menopausal or climacteric or "hot flash" or "hot flashes").ti,ab. | 124,663 |
|  | #12 | exp Menstrual Cycle/ or exp Menstruation Disturbances/ or (menstruation or menstruate or menstruates or menstruating or menstruated or menses or menstrual or dysmenorrhea or "painful period" or "painful periods" or "irregular period" or "irregular periods" or amenorrhea or menorrhagia or oligomenorrhea or premenstrual or pre-menstrual).ti,ab. | 97,232 |
|  | #13 | exp Urinary Tract Infections/ or exp Pelvic Floor Disorders/ or exp Polycystic Ovary Syndrome/ or exp Genital Neoplasms, Female/ or (PCOS or "polycystic ovary syndrome" or UTI or "urinary tract infection" or "urinary tract infections" or "pelvic floor disorder" or "pelvic floor disorders" or "disorders of the pelvic floor" or "pelvic floor health" or "pelvic floor prolapse").ti,ab. or ((ovary or ovaries or ovarian or uterine or uterus or cervical or cervix or vaginal or vagina or vaginas or fallopian or fallopians or endometrial or endometrium or endometriod or vulva or vulvas or vulvar) adj3 (cancer or cancers or cancerous or adenocarcinoma or adenocarcinomas or tumor or tumour or tumors or tumours or malignancy or malignancies or malignant or metastasis or metastases or metastasize or metastasizes or metastatic or neoplasm or neoplasms or cyst or cysts)).ti,ab. | 413,999 |
|  | #14 | exp Domestic Violence/ or exp Intimate Partner Violence/ or exp Spousal Abuse/or exp Battered Women/ or exp Rape/ or ((sex or sexual or sexually or domestic or partner or spouse or spousal or physical or physically) adj3 (abuse or abuses or abused or abuser or abusers or abusive or violence or violent or assault or assaults or assaulted)).ti,ab. or (rape or rapes or raped).ti,ab. | 85,126 |
| *combining* | #15 | 7 or 8 or 9 or 10 or 11 or 12 or 13 or 14 | 4,251,803 |
| *Qualitative study filter* | #16 | exp Qualitative Research/ or exp Focus Groups/ or exp Interviews as Topic/ or (qualitative or qualitatively or "focus group" or "focus groups" or "group discussion" or "group discussions" or ethnograph or ethnographic or ethnography or ethnographies or autoethnography or autoethnographies or autoethnographic or "key informant" or "lived experience" or "lived experiences" or phenomenology or phenomenological or "mixed method" or "mixed methods" or mixed-methods or mixed-method).ti,ab. or ((semi-structured or semistructured or in-depth or indepth) adj5 (interview or interviews or interviewed or interviewing or discussion or discussions)).ti,ab. or ((stakeholder or stakeholders) adj2 (interview or interviews or interviewed or interviewing or discussion or discussions)).ti,ab. or (thematic adj2 (analysis or analyses)).ti,ab. | 511,942 |
| *combining* | #17 | 6 and 15 and 16 | 3,131 |
| *Exclusions – study designs* | #18 | 17 not (case reports OR editorial OR letter OR comment OR congress).pt. | 3,107 |
| *Exclusions – animal-only research* | #19 | 18 not (exp animals/ not exp humans/) | 3,105 |
| *Date Limit* | #20 | Limit 19 to da=20100101-20230101 | 2,655 |
| *Language Limit* | #21 | 20 and English.lg. | 2,617 |

**Database: Embase (via Elsevier)**
Search Date: 10/10/2022
*Note: search from the Results page*

| **Description** | **Search Set** | **Search Strategy** | **Results** |
| --- | --- | --- | --- |
| *Virtual Care terms* | #1 | 'telemedicine'/exp OR 'teleconsultation'/exp OR 'videoconferencing'/exp OR 'telephone'/exp OR 'mobile phone'/exp OR 'smartphone'/exp | 147,860 |
|  | #2 | (telehealth OR tele-health OR telemedicine OR 'tele medicine' OR telemedical OR tele-medical OR telecare OR tele-care OR teleprimary care OR tele-primary care OR tele-PCP OR tele-visit OR televisit OR tele-visits OR televisits OR teleconference OR tele-conference OR teleconferences OR tele-conferences OR telemanage OR tele-manage OR telemanagement OR tele-management OR telepharmacy OR tele-pharmacy OR telepharmacies OR tele-pharmacies OR telepharmacist OR telepharmacists OR tele-pharmacist OR tele-pharmacists OR telegynecology OR tele-gynecology OR telegynecologist OR tele-gynecologist OR telegynecologists OR tele-gynecologists OR teleobstetrics OR tele-obstetrics OR teleobstetrician OR tele-obstetrician OR teleobstetricians OR tele-obstetricianstelenurse OR tele-nurse OR tele-nurses OR tele-nursing OR telenurse OR telenurses OR telenursing OR telepsychiatrist OR tele-psychiatrist OR telepsychiatrists OR tele-psychiatrists OR telepsychiatry OR tele-psychiatry OR telecounselling OR tele-counselling OR telecounselor OR tele-counselor OR teleconselors OR tele-counselors OR teleintervention OR tele-interventions OR teleinterventions OR tele-interventions OR tele-therapy OR tele-therapies OR teletherapy OR teletherapies):ti,ab | 25,512 |
|  | #3 | ((virtual OR virtually OR video OR video-based OR videobased OR videoconference OR video-conference OR videoconferences OR video-conferences OR videoconferencing OR video-conferencing OR webconference OR web-conference OR webconferences OR web-conferences OR webconferencing OR web-conferencing OR Zoom OR Skype OR WebEx OR FaceTime OR GoToMeeting OR 'web based' OR web-based OR webbased OR online OR telephone OR tele-phone OR telephones OR tele-phones OR telephoned OR tele-phoned OR cellphone OR cellphones OR cell-phone OR cell-phones OR 'cell phone' OR 'cell phones' OR smartphone OR smartphones OR smart-phone OR smart-phones OR 'smart phone' OR 'smart phones' OR 'cellular phone' OR 'cellular phones' OR 'mobile device' OR 'mobile devices' OR 'mobile phone' OR 'mobile phones' OR iPhone OR iPhones OR iPad OR iPads OR Android) NEAR/5 (care OR cared OR health OR healthcare OR appointment OR appointments OR meeting OR meetings OR met OR meet OR visit OR visits OR clinic OR clinics OR medicine OR medical OR therapy OR therapies OR therapeutic OR therapeutics OR intervention OR interventions OR treatment OR treatments OR treat OR treats OR treated OR manage OR manages OR management OR managed OR physician OR physicians OR clinician OR clinicians OR doctor OR doctors OR patient OR patients OR nurse OR nurses OR nursing OR diagnose OR diagnosis OR diagnoses OR diagnostic OR prescribe OR prescribes OR prescribing OR prescription OR prescriptions OR pharmacy OR pharmacies OR pharmacist OR pharmacists OR counsel OR counsels OR counselled OR counselling OR counsellor OR counsellors OR psychiatry OR psychiatrist OR psychiatrists OR 'mental health' OR gynecology OR gynecologist OR gynecologists OR obstetrics OR obstetrician OR obstetricians OR 'OB/GYN' OR OBGYN OR 'OB GYN')):ti,ab | 153,286 |
|  | #4 | (Tele NEAR/2 (care OR cared OR health OR healthcare OR appointment OR appointments OR meeting OR meetings OR visit OR visits OR clinic OR clinics OR medicine OR medical OR session OR sessions OR therapy OR therapies OR therapeutic OR therapeutics OR intervention OR interventions OR treatment OR treatments OR treat OR treats OR treated OR manage OR manages OR management OR managed OR physician OR physicians OR clinician OR clinicians OR doctor OR doctors OR nurse OR nurses OR nursing OR diagnose OR diagnosis OR diagnoses OR diagnostic OR prescribe OR prescribes OR prescribed OR prescribing OR prescription OR prescriptions OR pharmacy OR pharmacies OR pharmacist OR pharmacists OR psychiatry OR psychiatrists OR psychiatrist OR 'mental health' OR counselling OR counsel OR counsels OR counselled OR counsellor OR counsellors OR gynecology OR gynecologist OR gynecologists OR obstetrics OR obstetrician OR obstetricians OR 'OB/GYN' OR OBGYN OR 'OB GYN')):ti,ab | 2,350 |
|  | #5 | ((remote OR remotely) NEAR/3 (care OR cared OR health OR healthcare OR appointment OR appointments OR meeting OR meetings OR visit OR visits OR therapy OR therapies OR therapeutic OR therapeutics OR intervention OR interventions OR treatment OR treatments OR treat OR treats OR treated OR management OR managed OR diagnose OR diagnosis OR diagnoses OR diagnostic OR prescribe OR prescribes OR prescribed OR prescribing OR prescription OR prescriptions OR 'mental health' OR counselling OR counsels OR counselled OR counsel OR gynecology OR gynecologist OR gynecologists OR obstetrics OR obstetrician OR obstetricians OR 'OB/GYN' OR OBGYN OR 'OB GYN')):ti,ab | 10,361 |
| *combining* | #6 | #1 OR #2 OR #3 OR #4 OR #5 | 279,690 |
| *Women or women's health terms* | #7 | 'women`s health'/exp OR 'homosexual female'/exp OR 'male to female transgender'/exp OR (woman OR women OR womens OR womans OR 'women s' OR 'woman s' OR Female OR females OR 'female s' OR transwoman OR trans-woman OR transwomans OR 'transwoman s' OR trans-womans OR 'trans-woman s' OR trans-women OR transwomen OR transwomens OR 'transwomen s' OR trans-womens OR 'transwomen s' OR non-binary OR nonbinary OR transman OR trans-man OR transmans OR 'transman s' OR trans-mans OR 'trans-man s' OR transmen OR trans-men OR transmens OR trans-mens OR 'transmen s' OR 'transmen s' OR trans OR transgender OR transgendered OR lesbian OR lesbians):ti,ab | 3,670,009 |
|  | #8 | 'breast disease'/exp OR 'breast cancer'/exp OR ('breast health' OR 'breast disease' OR 'breast diseases' OR 'breast cancer' OR 'breast cancers' OR 'breast neoplasm' OR 'breast neoplasms'):ti,ab | 736,743 |
|  | #9 | 'pregnant woman'/exp OR 'maternal welfare'/exp OR 'maternal health service'/exp OR 'prenatal care'/exp OR 'postnatal care'/de OR 'maternal care'/exp OR 'perinatal care'/exp OR 'postnatal depression'/exp OR 'pregnancy'/exp OR 'pregnancy complication'/exp OR 'breast feeding'/exp OR 'lactation'/exp OR 'lactation disorder'/exp OR (pregnancy OR pregnancies OR pregnant OR pregnancy-induced OR pregnancy-associated OR prenatal OR pre-natal OR prenatally OR pre-natally OR perinatal OR peri-natal OR perinatally OR peri-natally OR postnatal OR post-natal OR postnatally OR post-natally OR maternal OR maternally OR postpartum OR post-partum OR breastfeeding OR 'breast feeding' OR lactation):ti,ab | 1,682,625 |
|  | #10 | 'contraception'/exp OR 'hormonal contraception'/exp OR 'oral contraceptive agent'/exp OR 'female contraceptive device'/exp OR 'prepregnancy care'/exp OR 'family planning'/exp OR 'female fertility'/exp OR 'female infertility'/exp OR 'fertility clinic'/exp OR 'abortion'/exp OR 'levonorgestrel'/exp OR (conception OR preconception OR pre-conception OR 'reproductive health' OR 'reproductive care' OR 'reproductive healthcare' OR 'reproductive plan' OR 'reproductive planning' OR 'family planning' OR fertility OR infertility OR contraception OR contraceptive OR contraceptives OR 'morning after pill' OR 'morning after pills' OR levonorgestrel OR 'plan b' OR abortifacient OR abortifacients OR misoprostol OR mifepristone OR RU-486 OR abortion OR abortions):ti,ab | 668,870 |
|  | #11 | 'menopause AND climacterium'/exp OR (menopause OR menopausal OR perimenopause OR peri-menopause OR perimenopausal OR peri-menopausal OR premenopause OR pre-menopause OR premenopausal OR pre-menopausal OR postmenopause OR post-menopause OR postmenopausal OR post-menopausal OR climacteric OR 'hot flash' OR 'hot flashes'):ti,ab | 200,889 |
|  | #12 | 'menstrual cycle'/exp OR 'menstruation disorder'/exp OR (menstruation OR menstruate OR menstruates OR menstruating OR menstruated OR menses OR menstrual OR dysmenorrhea OR 'painful period' OR 'painful periods' OR 'irregular period' OR 'irregular periods' OR amenorrhea OR menorrhagia OR oligomenorrhea OR premenstrual OR pre-menstrual):ti,ab | 234,478 |
|  | #13 | 'urinary tract infection'/exp OR 'pelvic floor disorder'/exp OR 'ovary polycystic disease'/exp OR 'female genital tract tumor'/exp OR (PCOS OR 'polycystic ovary syndrome' OR UTI OR 'urinary tract infection' OR 'urinary tract infections' OR 'pelvic floor disorder' OR 'pelvic floor disorders' OR 'disorders of the pelvic floor' OR 'pelvic floor health' OR 'pelvic floor prolapse'):ti,ab OR ((ovary OR ovaries OR ovarian OR uterine OR uterus OR cervical OR cervix OR vaginal OR vagina OR vaginas OR fallopian OR fallopians OR endometrial OR endometrium OR endometriod OR vulva OR vulvas OR vulvar) NEAR/3 (cancer OR cancers OR cancerous OR adenocarcinoma OR adenocarcinomas OR tumor OR tumour OR tumors OR tumours OR malignancy OR malignancies OR malignant OR metastasis OR metastases OR metastasize OR metastasizes OR metastatic OR neoplasm OR neoplasms OR cyst OR cysts)):ti,ab | 697,345 |
|  | #14 | 'domestic violence'/de OR 'battered woman'/exp OR 'family violence'/exp OR 'partner violence'/exp OR 'rape'/exp OR ((sex OR sexual OR sexually OR domestic OR partner OR spouse OR spousal OR physical OR physically) NEAR/3 (abuse OR abuses OR abused OR abuser OR abusers OR abusive OR violence OR violent OR assault OR assaults OR assaulted)):ti,ab OR (rape OR rapes OR raped):ti,ab | 79,413 |
| *combining* | #15 | #7 OR #8 OR #9 OR #10 OR #11 OR #12 OR #13 OR #14 | 6,089,360 |
| *Qualitative study filter* | #16 | 'qualitative research'/exp OR 'interview'/de OR 'audio interview'/exp OR 'semi structured interview'/exp OR 'structured interview'/exp OR 'telephone interview'/de OR 'video interview'/exp OR (qualitative OR qualitatively OR 'focus group' OR 'focus groups' OR 'group discussion' OR 'group discussions' OR ethnograph OR ethnographic OR ethnography OR ethnographies OR autoethnography OR autoethnographies OR autoethnographic OR 'key informant' OR 'lived experience' OR 'lived experiences' OR phenomenology OR phenomenological OR 'mixed method' OR 'mixed methods' OR mixed-methods OR mixed-method):ti,ab OR ((semi-structured OR semistructured OR in-depth OR indepth) NEAR/5 (interview OR interviews OR interviewed OR interviewing OR discussion OR discussions)):ti,ab OR ((stakeholder OR stakeholders) NEAR/2 (interview OR interviews OR interviewed OR interviewing OR discussion OR discussions)):ti,ab OR (thematic NEAR/2 (analysis OR analyses)):ti,ab | 784,781 |
| *combining* | #17 | #6 AND #15 AND #16 | 9,039 |
| *Exclusions – study designs* | #18 | #17 NOT ('case report'/exp OR 'case study'/exp OR 'editorial'/exp OR [editorial]/lim OR 'letter'/exp OR [letter]/lim OR 'note'/exp OR [note]/lim OR [conference abstract]/lim OR 'conference abstract'/exp OR 'conference abstract'/it) | 5,028 |
| *Exclusions – animal-only research* | #19 | #18 AND [humans]/lim | 4,919 |
| *Date Limit* | #20 | #19 AND [01-01-2010]/sd | 3,621 |
| *Language Limit* | #21 | #20 AND [english]/lim | 3,565 |

**Database: CINAHL Complete (via EBSCO)**
Search date: 10/10/2022

| **Description** | **Search Set** | **Search Strategy** | **Results** |
| --- | --- | --- | --- |
| *Virtual Care terms* | #1 | (MH "Telemedicine+") OR (MH "Remote Consultation") OR (MH "Videoconferencing+") OR (MH "Telephone+") OR (MH "Cellular Phone+") OR (MH "Smartphone") | 49,564 |
|  | #2 | ((TI telehealth OR AB telehealth) OR (TI tele-health OR AB tele-health) OR (TI telemedicine OR AB telemedicine) OR (TI tele-medicine OR AB tele-medicine) OR (TI telemedical OR AB telemedical) OR (TI tele-medical OR AB tele-medical) OR (TI telecare OR AB telecare) OR (TI tele-care OR AB tele-care) OR (TI "teleprimary care" OR AB "teleprimary care") OR (TI "tele-primary care" OR AB "tele-primary care") OR (TI tele-PCP OR AB tele-PCP) OR (TI tele-visit OR AB tele-visit) OR (TI televisit OR AB televisit) OR (TI tele-visits OR AB tele-visits) OR (TI televisits OR AB televisits) OR (TI teleconference OR AB teleconference) OR (TI tele-conference OR AB tele-conference) OR (TI teleconferences OR AB teleconferences) OR (TI tele-conferences OR AB tele-conferences) OR (TI telemanage OR AB telemanage) OR (TI tele-manage OR AB tele-manage) OR (TI telemanagement OR AB telemanagement) OR (TI tele-management OR AB tele-management) OR (TI telepharmacy OR AB telepharmacy) OR (TI tele-pharmacy OR AB tele-pharmacy) OR (TI telepharmacies OR AB telepharmacies) OR (TI tele-pharmacies OR AB tele-pharmacies) OR (TI telepharmacist OR AB telepharmacist) OR (TI telepharmacists OR AB telepharmacists) OR (TI tele-pharmacist OR AB tele-pharmacist) OR (TI tele-pharmacists OR AB tele-pharmacists) OR (TI telegynecology OR AB telegynecology) OR (TI tele-gynecology OR AB tele-gynecology) OR (TI telegynecologist OR AB telegynecologist) OR (TI tele-gynecologist OR AB tele-gynecologist) OR (TI telegynecologists OR AB telegynecologists) OR (TI tele-gynecologists OR AB tele-gynecologists) OR (TI teleobstetrics OR AB teleobstetrics) OR (TI tele-obstetrics OR AB tele-obstetrics) OR (TI teleobstetrician OR AB teleobstetrician) OR (TI tele-obstetrician OR AB tele-obstetrician) OR (TI teleobstetricians OR AB teleobstetricians) OR (TI tele-obstetricianstelenurse OR AB tele-obstetricianstelenurse) OR (TI tele-nurse OR AB tele-nurse) OR (TI tele-nurses OR AB tele-nurses) OR (TI tele-nursing OR AB tele-nursing) OR (TI telenurse OR AB telenurse) OR (TI telenurses OR AB telenurses) OR (TI telenursing OR AB telenursing) OR (TI telepsychiatrist OR AB telepsychiatrist) OR (TI tele-psychiatrist OR AB tele-psychiatrist) OR (TI telepsychiatrists OR AB telepsychiatrists) OR (TI tele-psychiatrists OR AB tele-psychiatrists) OR (TI telepsychiatry OR AB telepsychiatry) OR (TI tele-psychiatry OR AB tele-psychiatry) OR (TI telecounselling OR AB telecounselling) OR (TI tele-counselling OR AB tele-counselling) OR (TI telecounselor OR AB telecounselor) OR (TI tele-counselor OR AB tele-counselor) OR (TI teleconselors OR AB teleconselors) OR (TI tele-counselors OR AB tele-counselors) OR (TI teleintervention OR AB teleintervention) OR (TI tele-interventions OR AB tele-interventions) OR (TI teleinterventions OR AB teleinterventions) OR (TI tele-interventions OR AB tele-interventions) OR (TI tele-therapy OR AB tele-therapy) OR (TI tele-therapies OR AB tele-therapies) OR (TI teletherapy OR AB teletherapy) OR (TI teletherapies OR AB teletherapies)) | 16,334 |
|  | #3 | (((TI virtual OR AB virtual) OR (TI virtually OR AB virtually) OR (TI video OR AB video) OR (TI video-based OR AB video-based) OR (TI videobased OR AB videobased) OR (TI videoconference OR AB videoconference) OR (TI video-conference OR AB video-conference) OR (TI videoconferences OR AB videoconferences) OR (TI video-conferences OR AB video-conferences) OR (TI videoconferencing OR AB videoconferencing) OR (TI video-conferencing OR AB video-conferencing) OR (TI webconference OR AB webconference) OR (TI web-conference OR AB web-conference) OR (TI webconferences OR AB webconferences) OR (TI web-conferences OR AB web-conferences) OR (TI webconferencing OR AB webconferencing) OR (TI web-conferencing OR AB web-conferencing) OR (TI Zoom OR AB Zoom) OR (TI Skype OR AB Skype) OR (TI WebEx OR AB WebEx) OR (TI FaceTime OR AB FaceTime) OR (TI GoToMeeting OR AB GoToMeeting) OR (TI "web based" OR AB "web based") OR (TI web-based OR AB web-based) OR (TI webbased OR AB webbased) OR (TI online OR AB online) OR (TI telephone OR AB telephone) OR (TI tele-phone OR AB tele-phone) OR (TI telephones OR AB telephones) OR (TI tele-phones OR AB tele-phones) OR (TI telephoned OR AB telephoned) OR (TI tele-phoned OR AB tele-phoned) OR (TI cellphone OR AB cellphone) OR (TI cellphones OR AB cellphones) OR (TI cell-phone OR AB cell-phone) OR (TI cell-phones OR AB cell-phones) OR (TI "cell phone" OR AB "cell phone") OR (TI "cell phones" OR AB "cell phones") OR (TI smartphone OR AB smartphone) OR (TI smartphones OR AB smartphones) OR (TI smart-phone OR AB smart-phone) OR (TI smart-phones OR AB smart-phones) OR (TI "smart phone" OR AB "smart phone") OR (TI "smart phones" OR AB "smart phones") OR (TI "cellular phone" OR AB "cellular phone") OR (TI "cellular phones" OR AB "cellular phones") OR (TI "mobile device" OR AB "mobile device") OR (TI "mobile devices" OR AB "mobile devices") OR (TI "mobile phone" OR AB "mobile phone") OR (TI "mobile phones" OR AB "mobile phones") OR (TI iPhone OR AB iPhone) OR (TI iPhones OR AB iPhones) OR (TI iPad OR AB iPad) OR (TI iPads OR AB iPads) OR (TI Android OR AB Android)) N5 ((TI care OR AB care) OR (TI cared OR AB cared) OR (TI health OR AB health) OR (TI healthcare OR AB healthcare) OR (TI appointment OR AB appointment) OR (TI appointments OR AB appointments) OR (TI meeting OR AB meeting) OR (TI meetings OR AB meetings) OR (TI met OR AB met) OR (TI meet OR AB meet) OR (TI visit OR AB visit) OR (TI visits OR AB visits) OR (TI clinic OR AB clinic) OR (TI clinics OR AB clinics) OR (TI medicine OR AB medicine) OR (TI medical OR AB medical) OR (TI therapy OR AB therapy) OR (TI therapies OR AB therapies) OR (TI therapeutic OR AB therapeutic) OR (TI therapeutics OR AB therapeutics) OR (TI intervention OR AB intervention) OR (TI interventions OR AB interventions) OR (TI treatment OR AB treatment) OR (TI treatments OR AB treatments) OR (TI treat OR AB treat) OR (TI treats OR AB treats) OR (TI treated OR AB treated) OR (TI manage OR AB manage) OR (TI manages OR AB manages) OR (TI management OR AB management) OR (TI managed OR AB managed) OR (TI physician OR AB physician) OR (TI physicians OR AB physicians) OR (TI clinician OR AB clinician) OR (TI clinicians OR AB clinicians) OR (TI doctor OR AB doctor) OR (TI doctors OR AB doctors) OR (TI patient OR AB patient) OR (TI patients OR AB patients) OR (TI nurse OR AB nurse) OR (TI nurses OR AB nurses) OR (TI nursing OR AB nursing) OR (TI diagnose OR AB diagnose) OR (TI diagnosis OR AB diagnosis) OR (TI diagnoses OR AB diagnoses) OR (TI diagnostic OR AB diagnostic) OR (TI prescribe OR AB prescribe) OR (TI prescribes OR AB prescribes) OR (TI prescribing OR AB prescribing) OR (TI prescription OR AB prescription) OR (TI prescriptions OR AB prescriptions) OR (TI pharmacy OR AB pharmacy) OR (TI pharmacies OR AB pharmacies) OR (TI pharmacist OR AB pharmacist) OR (TI pharmacists OR AB pharmacists) OR (TI counsel OR AB counsel) OR (TI counsels OR AB counsels) OR (TI counselled OR AB counselled) OR (TI counselling OR AB counselling) OR (TI counsellor OR AB counsellor) OR (TI counsellors OR AB counsellors) OR (TI psychiatry OR AB psychiatry) OR (TI psychiatrist OR AB psychiatrist) OR (TI psychiatrists OR AB psychiatrists) OR (TI "mental health" OR AB "mental health") OR (TI gynecology OR AB gynecology) OR (TI gynecologist OR AB gynecologist) OR (TI gynecologists OR AB gynecologists) OR (TI obstetrics OR AB obstetrics) OR (TI obstetrician OR AB obstetrician) OR (TI obstetricians OR AB obstetricians) OR (TI OB/GYN OR AB OB/GYN) OR (TI OBGYN OR AB OBGYN) OR (TI "OB GYN" OR AB "OB GYN"))) | 70,927 |
|  | #4 | ((TI Tele OR AB Tele) N2 ((TI care OR AB care) OR (TI cared OR AB cared) OR (TI health OR AB health) OR (TI healthcare OR AB healthcare) OR (TI appointment OR AB appointment) OR (TI appointments OR AB appointments) OR (TI meeting OR AB meeting) OR (TI meetings OR AB meetings) OR (TI visit OR AB visit) OR (TI visits OR AB visits) OR (TI clinic OR AB clinic) OR (TI clinics OR AB clinics) OR (TI medicine OR AB medicine) OR (TI medical OR AB medical) OR (TI session OR AB session) OR (TI sessions OR AB sessions) OR (TI therapy OR AB therapy) OR (TI therapies OR AB therapies) OR (TI therapeutic OR AB therapeutic) OR (TI therapeutics OR AB therapeutics) OR (TI intervention OR AB intervention) OR (TI interventions OR AB interventions) OR (TI treatment OR AB treatment) OR (TI treatments OR AB treatments) OR (TI treat OR AB treat) OR (TI treats OR AB treats) OR (TI treated OR AB treated) OR (TI manage OR AB manage) OR (TI manages OR AB manages) OR (TI management OR AB management) OR (TI managed OR AB managed) OR (TI physician OR AB physician) OR (TI physicians OR AB physicians) OR (TI clinician OR AB clinician) OR (TI clinicians OR AB clinicians) OR (TI doctor OR AB doctor) OR (TI doctors OR AB doctors) OR (TI nurse OR AB nurse) OR (TI nurses OR AB nurses) OR (TI nursing OR AB nursing) OR (TI diagnose OR AB diagnose) OR (TI diagnosis OR AB diagnosis) OR (TI diagnoses OR AB diagnoses) OR (TI diagnostic OR AB diagnostic) OR (TI prescribe OR AB prescribe) OR (TI prescribes OR AB prescribes) OR (TI prescribed OR AB prescribed) OR (TI prescribing OR AB prescribing) OR (TI prescription OR AB prescription) OR (TI prescriptions OR AB prescriptions) OR (TI pharmacy OR AB pharmacy) OR (TI pharmacies OR AB pharmacies) OR (TI pharmacist OR AB pharmacist) OR (TI pharmacists OR AB pharmacists) OR (TI psychiatry OR AB psychiatry) OR (TI psychiatrists OR AB psychiatrists) OR (TI psychiatrist OR AB psychiatrist) OR (TI "mental health" OR AB "mental health") OR (TI counselling OR AB counselling) OR (TI counsel OR AB counsel) OR (TI counsels OR AB counsels) OR (TI counselled OR AB counselled) OR (TI counsellor OR AB counsellor) OR (TI counsellors OR AB counsellors) OR (TI gynecology OR AB gynecology) OR (TI gynecologist OR AB gynecologist) OR (TI gynecologists OR AB gynecologists) OR (TI obstetrics OR AB obstetrics) OR (TI obstetrician OR AB obstetrician) OR (TI obstetricians OR AB obstetricians) OR (TI OB/GYN OR AB OB/GYN) OR (TI OBGYN OR AB OBGYN) OR (TI "OB GYN" OR AB "OB GYN"))) | 784 |
|  | #5 | (((TI remote OR AB remote) OR (TI remotely OR AB remotely)) N3 ((TI care OR AB care) OR (TI cared OR AB cared) OR (TI health OR AB health) OR (TI healthcare OR AB healthcare) OR (TI appointment OR AB appointment) OR (TI appointments OR AB appointments) OR (TI meeting OR AB meeting) OR (TI meetings OR AB meetings) OR (TI visit OR AB visit) OR (TI visits OR AB visits) OR (TI therapy OR AB therapy) OR (TI therapies OR AB therapies) OR (TI therapeutic OR AB therapeutic) OR (TI therapeutics OR AB therapeutics) OR (TI intervention OR AB intervention) OR (TI interventions OR AB interventions) OR (TI treatment OR AB treatment) OR (TI treatments OR AB treatments) OR (TI treat OR AB treat) OR (TI treats OR AB treats) OR (TI treated OR AB treated) OR (TI management OR AB management) OR (TI managed OR AB managed) OR (TI diagnose OR AB diagnose) OR (TI diagnosis OR AB diagnosis) OR (TI diagnoses OR AB diagnoses) OR (TI diagnostic OR AB diagnostic) OR (TI prescribe OR AB prescribe) OR (TI prescribes OR AB prescribes) OR (TI prescribed OR AB prescribed) OR (TI prescribing OR AB prescribing) OR (TI prescription OR AB prescription) OR (TI prescriptions OR AB prescriptions) OR (TI "mental health" OR AB "mental health") OR (TI counselling OR AB counselling) OR (TI counsels OR AB counsels) OR (TI counselled OR AB counselled) OR (TI counsel OR AB counsel) OR (TI gynecology OR AB gynecology) OR (TI gynecologist OR AB gynecologist) OR (TI gynecologists OR AB gynecologists) OR (TI obstetrics OR AB obstetrics) OR (TI obstetrician OR AB obstetrician) OR (TI obstetricians OR AB obstetricians) OR (TI OB/GYN OR AB OB/GYN) OR (TI OBGYN OR AB OBGYN) OR (TI "OB GYN" OR AB "OB GYN"))) | 4084 |
| *combining* | #6 | S1 OR S2 OR S3 OR S4 OR S5 | 121,480 |
| *Women OR women's health terms* | #7 | (MH "Women+") OR (MH "Women's Health") OR (MH "Trans Women") OR (MH "Lesbians") OR ((TI woman OR AB woman) OR (TI women OR AB women) OR (TI womens OR AB womens) OR (TI womans OR AB womans) OR (TI "women s" OR AB "women s") OR (TI "woman s" OR AB "woman s") OR (TI Female OR AB Female) OR (TI females OR AB females) OR (TI "female s" OR AB "female s") OR (TI transwoman OR AB transwoman) OR (TI trans-woman OR AB trans-woman) OR (TI transwomans OR AB transwomans) OR (TI "transwoman s" OR AB "transwoman s") OR (TI trans-womans OR AB trans-womans) OR (TI "trans-woman s" OR AB "trans-woman s") OR (TI trans-women OR AB trans-women) OR (TI transwomen OR AB transwomen) OR (TI transwomens OR AB transwomens) OR (TI "transwomen s" OR AB "transwomen s") OR (TI trans-womens OR AB trans-womens) OR (TI "transwomen s" OR AB "transwomen s") OR (TI non-binary OR AB non-binary) OR (TI nonbinary OR AB nonbinary) OR (TI transman OR AB transman) OR (TI trans-man OR AB trans-man) OR (TI transmans OR AB transmans) OR (TI "transman s" OR AB "transman s") OR (TI trans-mans OR AB trans-mans) OR (TI "trans-man s" OR AB "trans-man s") OR (TI transmen OR AB transmen) OR (TI trans-men OR AB trans-men) OR (TI transmens OR AB transmens) OR (TI trans-mens OR AB trans-mens) OR (TI "transmen s" OR AB "transmen s") OR (TI "transmen s" OR AB "transmen s") OR (TI trans OR AB trans) OR (TI transgender OR AB transgender) OR (TI transgendered OR AB transgendered) OR (TI lesbian OR AB lesbian) OR (TI lesbians OR AB lesbians)) | 725,847 |
|  | #8 | (MH "Breast Diseases+") OR (MH "Breast Neoplasms+") OR ((TI "breast health" OR AB "breast health") OR (TI "breast disease" OR AB "breast disease") OR (TI "breast diseases" OR AB "breast diseases") OR (TI "breast cancer" OR AB "breast cancer") OR (TI "breast cancers" OR AB "breast cancers") OR (TI "breast neoplasm" OR AB "breast neoplasm") OR (TI "breast neoplasms" OR AB "breast neoplasms")) | 117,351 |
|  | #9 | (MH "Expectant Mothers") OR (MH "Maternal Health Services+") OR (MH "Prenatal Care") OR (MH "Perinatal Care") OR (MH "Postnatal Care+") OR (MH "Postnatal Period+") OR (MH "Depression, Postpartum") OR (MH "Pregnancy+") OR (MH "Pregnancy Complications+") OR (MH "Breast Feeding+") OR (MH "Lactation") OR (MH "Lactation Disorders+") OR ((TI pregnancy OR AB pregnancy) OR (TI pregnancies OR AB pregnancies) OR (TI pregnant OR AB pregnant) OR (TI pregnancy-induced OR AB pregnancy-induced) OR (TI pregnancy-associated OR AB pregnancy-associated) OR (TI prenatal OR AB prenatal) OR (TI pre-natal OR AB pre-natal) OR (TI prenatally OR AB prenatally) OR (TI pre-natally OR AB pre-natally) OR (TI perinatal OR AB perinatal) OR (TI peri-natal OR AB peri-natal) OR (TI perinatally OR AB perinatally) OR (TI peri-natally OR AB peri-natally) OR (TI postnatal OR AB postnatal) OR (TI post-natal OR AB post-natal) OR (TI postnatally OR AB postnatally) OR (TI post-natally OR AB post-natally) OR (TI maternal OR AB maternal) OR (TI maternally OR AB maternally) OR (TI postpartum OR AB postpartum) OR (TI post-partum OR AB post-partum) OR (TI breastfeeding OR AB breastfeeding) OR (TI "breast feeding" OR AB "breast feeding") OR (TI lactation OR AB lactation)) | 388,396 |
|  | #10 | (MH "Contraception+") OR (MH "Hormonal Contraception") OR (MH "Contraceptives, Postcoital+") OR (MH "Contraceptives, Oral+") OR (MH "Contraceptive Devices+") OR (MH "Prepregnancy Care") OR (MH "Fertility+") OR (MH "Infertility+") OR (MH "Fertility Clinics") OR (MH "Abortion, Incomplete") OR (MH "Abortion, Induced+") OR (MH "Abortion, Spontaneous+") OR (MH "Levonorgestrel") OR ((TI conception OR AB conception) OR (TI preconception OR AB preconception) OR (TI pre-conception OR AB pre-conception) OR (TI "reproductive health" OR AB "reproductive health") OR (TI "reproductive care" OR AB "reproductive care") OR (TI "reproductive healthcare" OR AB "reproductive healthcare") OR (TI "reproductive plan" OR AB "reproductive plan") OR (TI "reproductive planning" OR AB "reproductive planning") OR (TI "family planning" OR AB "family planning") OR (TI fertility OR AB fertility) OR (TI infertility OR AB infertility) OR (TI contraception OR AB contraception) OR (TI contraceptive OR AB contraceptive) OR (TI contraceptives OR AB contraceptives) OR (TI "morning after pill" OR AB "morning after pill") OR (TI "morning after pills" OR AB "morning after pills") OR (TI levonorgestrel OR AB levonorgestrel) OR (TI "plan b" OR AB "plan b") OR (TI abortifacient OR AB abortifacient) OR (TI abortifacients OR AB abortifacients) OR (TI misoprostol OR AB misoprostol) OR (TI mifepristone OR AB mifepristone) OR (TI RU-486 OR AB RU-486) OR (TI abortion OR AB abortion) OR (TI abortions OR AB abortions)) | 117,151 |
|  | #11 | (MH "Menopause+") OR ((TI menopause OR AB menopause) OR (TI menopausal OR AB menopausal) OR (TI perimenopause OR AB perimenopause) OR (TI peri-menopause OR AB peri-menopause) OR (TI perimenopausal OR AB perimenopausal) OR (TI peri-menopausal OR AB peri-menopausal) OR (TI premenopause OR AB premenopause) OR (TI pre-menopause OR AB pre-menopause) OR (TI premenopausal OR AB premenopausal) OR (TI pre-menopausal OR AB pre-menopausal) OR (TI postmenopause OR AB postmenopause) OR (TI post-menopause OR AB post-menopause) OR (TI postmenopausal OR AB postmenopausal) OR (TI post-menopausal OR AB post-menopausal) OR (TI climacteric OR AB climacteric) OR (TI "hot flash" OR AB "hot flash") OR (TI "hot flashes" OR AB "hot flashes")) | 41,218 |
|  | #12 | (MH "Menstrual Cycle+") OR (MH "Menstruation Disorders+") OR ((TI menstruation OR AB menstruation) OR (TI menstruate OR AB menstruate) OR (TI menstruates OR AB menstruates) OR (TI menstruating OR AB menstruating) OR (TI menstruated OR AB menstruated) OR (TI menses OR AB menses) OR (TI menstrual OR AB menstrual) OR (TI dysmenorrhea OR AB dysmenorrhea) OR (TI "painful period" OR AB "painful period") OR (TI "painful periods" OR AB "painful periods") OR (TI "irregular period" OR AB "irregular period") OR (TI "irregular periods" OR AB "irregular periods") OR (TI amenorrhea OR AB amenorrhea) OR (TI menorrhagia OR AB menorrhagia) OR (TI oligomenorrhea OR AB oligomenorrhea) OR (TI premenstrual OR AB premenstrual) OR (TI pre-menstrual OR AB pre-menstrual)) | 22,729 |
|  | #13 | (MH "Urinary Tract Infections+") OR (MH "Pelvic Floor Disorders") OR (MH "Polycystic Ovary Syndrome") OR (MH "Genital Neoplasms, Female+") OR ((TI PCOS OR AB PCOS) OR (TI "polycystic ovary syndrome" OR AB "polycystic ovary syndrome") OR (TI UTI OR AB UTI) OR (TI "urinary tract infection" OR AB "urinary tract infection") OR (TI "urinary tract infections" OR AB "urinary tract infections") OR (TI "pelvic floor disorder" OR AB "pelvic floor disorder") OR (TI "pelvic floor disorders" OR AB "pelvic floor disorders") OR (TI "disorders of the pelvic floor" OR AB "disorders of the pelvic floor") OR (TI "pelvic floor health" OR AB "pelvic floor health") OR (TI "pelvic floor prolapse" OR AB "pelvic floor prolapse")) OR (((TI ovary OR AB ovary) OR (TI ovaries OR AB ovaries) OR (TI ovarian OR AB ovarian) OR (TI uterine OR AB uterine) OR (TI uterus OR AB uterus) OR (TI cervical OR AB cervical) OR (TI cervix OR AB cervix) OR (TI vaginal OR AB vaginal) OR (TI vagina OR AB vagina) OR (TI vaginas OR AB vaginas) OR (TI fallopian OR AB fallopian) OR (TI fallopians OR AB fallopians) OR (TI endometrial OR AB endometrial) OR (TI endometrium OR AB endometrium) OR (TI endometriod OR AB endometriod) OR (TI vulva OR AB vulva) OR (TI vulvas OR AB vulvas) OR (TI vulvar OR AB vulvar)) N3 ((TI cancer OR AB cancer) OR (TI cancers OR AB cancers) OR (TI cancerous OR AB cancerous) OR (TI adenocarcinoma OR AB adenocarcinoma) OR (TI adenocarcinomas OR AB adenocarcinomas) OR (TI tumor OR AB tumor) OR (TI tumour OR AB tumour) OR (TI tumors OR AB tumors) OR (TI tumours OR AB tumours) OR (TI malignancy OR AB malignancy) OR (TI malignancies OR AB malignancies) OR (TI malignant OR AB malignant) OR (TI metastasis OR AB metastasis) OR (TI metastases OR AB metastases) OR (TI metastasize OR AB metastasize) OR (TI metastasizes OR AB metastasizes) OR (TI metastatic OR AB metastatic) OR (TI neoplasm OR AB neoplasm) OR (TI neoplasms OR AB neoplasms) OR (TI cyst OR AB cyst) OR (TI cysts OR AB cysts))) | 85,150 |
|  | #14 | (MH "Domestic Violence") OR (MH "Intimate Partner Violence") OR (MH "Battered Women") OR (MH "Rape") OR (((TI sex OR AB sex) OR (TI sexual OR AB sexual) OR (TI sexually OR AB sexually) OR (TI domestic OR AB domestic) OR (TI partner OR AB partner) OR (TI spouse OR AB spouse) OR (TI spousal OR AB spousal) OR (TI physical OR AB physical) OR (TI physically OR AB physically)) N3 ((TI abuse OR AB abuse) OR (TI abuses OR AB abuses) OR (TI abused OR AB abused) OR (TI abuser OR AB abuser) OR (TI abusers OR AB abusers) OR (TI abusive OR AB abusive) OR (TI violence OR AB violence) OR (TI violent OR AB violent) OR (TI assault OR AB assault) OR (TI assaults OR AB assaults) OR (TI assaulted OR AB assaulted))) OR ((TI rape OR AB rape) OR (TI rapes OR AB rapes) OR (TI raped OR AB raped)) | 46,224 |
| *combining* | #15 | S7 OR S8 OR S9 OR S10 OR S11 OR S12 OR S13 OR S14 | 1,161,334 |
| *Qualitative study filter* | #16 | (MH "Qualitative Studies+") OR (MH "Focus Groups") OR (MH "Interviews+") OR (MH "Semi-Structured Interview") OR (MH "Structured Interview") OR ((TI qualitative OR AB qualitative) OR (TI qualitatively OR AB qualitatively) OR (TI "focus group" OR AB "focus group") OR (TI "focus groups" OR AB "focus groups") OR (TI "group discussion" OR AB "group discussion") OR (TI "group discussions" OR AB "group discussions") OR (TI ethnograph OR AB ethnograph) OR (TI ethnographic OR AB ethnographic) OR (TI ethnography OR AB ethnography) OR (TI ethnographies OR AB ethnographies) OR (TI autoethnography OR AB autoethnography) OR (TI autoethnographies OR AB autoethnographies) OR (TI autoethnographic OR AB autoethnographic) OR (TI "key informant" OR AB "key informant") OR (TI "lived experience" OR AB "lived experience") OR (TI "lived experiences" OR AB "lived experiences") OR (TI phenomenology OR AB phenomenology) OR (TI phenomenological OR AB phenomenological) OR (TI "mixed method" OR AB "mixed method") OR (TI "mixed methods" OR AB "mixed methods") OR (TI mixed-methods OR AB mixed-methods) OR (TI mixed-method OR AB mixed-method)) OR (((TI semi-structured OR AB semi-structured) OR (TI semistructured OR AB semistructured) OR (TI in-depth OR AB in-depth) OR (TI indepth OR AB indepth)) N5 ((TI interview OR AB interview) OR (TI interviews OR AB interviews) OR (TI interviewed OR AB interviewed) OR (TI interviewing OR AB interviewing) OR (TI discussion OR AB discussion) OR (TI discussions OR AB discussions))) OR (((TI stakeholder OR AB stakeholder) OR (TI stakeholders OR AB stakeholders)) N2 ((TI interview OR AB interview) OR (TI interviews OR AB interviews) OR (TI interviewed OR AB interviewed) OR (TI interviewing OR AB interviewing) OR (TI discussion OR AB discussion) OR (TI discussions OR AB discussions))) OR ((TI thematic OR AB thematic) N2 ((TI analysis OR AB analysis) OR (TI analyses OR AB analyses))) | 423,110 |
| *combining* | #17 | S6 AND S15 AND S16 | 4,539 |
| *Exclusions – study designs* | #18 | S17 NOT PT ( Abstract OR Algorithm OR Anecdote OR Bibliography OR Book OR Book Chapter OR Book Review OR Brief Item OR Care Plan OR Cartoon OR Case Study OR CEU OR Code of Ethics OR Commentary OR Computer Program OR Critical Path OR Diagnostic Images OR Directories OR Doctoral Dissertation OR Editorial OR Games OR Glossary OR Letter OR Masters Thesis OR Obituary OR Pamphlet OR Pamphlet Chapter OR Poetry OR Proceedings) | 4,140 |
| *Exclusions – animal-only research* | #19 | S18 NOT (((MH "Animals+") OR (MH "Animal Studies") OR (TI "animal model*")) NOT (MH "human")) | 4,140 |
| *Date Limit* | #20 | Limiters - Published Date: 20100101-20221231 | 2,663 |
| *Language Limit* | #21 | Limiters – English language | 2,636 |

**Appendix B. Eligibility Criteria for Qualitative Evidence Synthesis**

| SPIDER Eligibility criteria | | |
| --- | --- | --- |
| Study Characteristic | Inclusion Criteria | Exclusion Criteria |
| Sample | - Individuals identifying as women/female who have been offered or received synchronous clinical care via a virtual modality for any condition (e.g., gender-specific or gender-neutral) - Clinical team members involved in providing virtual care to individuals identifying as women or female - *Virtual modalities* include clinical care provision delivered synchronously (i.e., in real time) between a patient(s) and clinical team member specifically via video-based conferencing or telephone - *Clinical care provision* to include 1:1 or group-based encounters delivered as part of a clinic setting and which is provided by or consulted to by a treating clinician - *Clinical team members* include any professional role involved in delivering clinic-based healthcare or coordinating/scheduling of clinic-based care, including administrative staff (e.g., management, clinic-based administrative staff), nursing, prescribing-providers - Focus on parent as patient (e.g., lactation consultation) | - Men or individuals identifying as male - Mixed gender populations for which the results are not stratified, or intervention is not subgrouped by gender - Virtual care modalities for asynchronous (i.e., not in real time) including secure messaging, static health related webpages, store, and forward services - Text-based chat – even if synchronous - Clinician to clinician virtual communication that does not include patient participation (e.g., e-consults) - Non-specific peer led support groups - Sample exclusively under 18, or mixed age range with a mean or standard deviation under 18 - Focus on infant or child rather than parent as patient |
| Phenomenon of Interest | - Virtual care delivered remotely (i.e., patient and provider in separate physical locations) and which involves the transmission of clinical information from patient to clinical team synchronously. Bidirectional information exchange between provider and clinician (e.g., not a health education workshop led by a clinician) - Care provided by the VA already (e.g., lactation consultation) - Clinician experiences delivering care to women digitally - Studies that include novel interventions, pilots, and usual care that focuses on the actual experiences of women using telehealth, rather than perceived/anticipated experiences. We will include articles that assess the feasibility of a virtual modality for usual care as well as articles about women who sought/want telehealth but were unable to receive telehealth (e.g., due to poor fit, lack of access, etc.) - Studies about interpersonal violence (IPV), domestic violence (DV), or sexual assault (SA) that specifically focus on these topics as experienced by women or gender diverse people | - Self-management interventions that do not involve communication with a clinical team member or do not involve a patient sharing some of their own information - Virtual healthcare that is asynchronous as described above - Non-specific peer support groups - Support delivered unrelated to specific clinical conditions (e.g., benefits process support, training on financial literacy) - Mobile App interventions or any interventions where the virtual care part is not required or the dominant part - Care not provided by the VA (e.g., neonatal care) - Articles about clinicians that do not pertain to delivery of care to women digitally (e.g., wellbeing of clinicians) - Articles about the feasibility of novel interventions or potential experiences of a novel intervention that happens to have a virtual modality - Studies about interpersonal violence (IPV), domestic violence (DV), or sexual assault (SA) that **do not** specifically focus on these topics as experienced by women or gender diverse people |
| Design | - Synchronous data collection gathered via interviews (individual, dyad, group; semi-structured or structured) - Focus groups; observations; ethnographies | - Not open-ended surveys or asynchronous means of collecting data - Content analysis - Protocol papers - Systematic reviews and scoping reviews (tag for later review if relevant, however) |
| Evaluation | Primary purpose of data collection is to evaluate the experiences and perspectives around being offered or receiving virtual care for women or the delivery of synchronous virtual care to individuals who identify as women or female | |
| Research Type | Qualitative or mixed or multiple methods | Quantitative-only study designs |
| Countries | [Organization for Economic Co-operation and Development (OECD) Member Countries](https://www.oecd.org/about/document/ratification-oecd-convention.htm) | Non-OECD |
| Time | Publications starting January 1^st^, 2010 | Publications from December 31, 2009, or earlier |
| Publications | Full, peer-reviewed manuscripts | Posters, abstracts, letters |

Appendix C

| **Qualitative Interview Guide Domains and Example Questions** | |
| --- | --- |
| **Interview Domain** | **Question Examples*** |
| **Women’s Health Conditions** | Patients: Please tell us for which health condition(s) you are most comfortable using phone or video care in VA.  Providers: For which health care issues do you feel virtual care is appropriate for women in the VA? |
| **Technology of  Virtual Care** | Patients: What supports do you need to engage with technology for video visits?  Providers: What are the key technologic aspects of delivering care to women over video visits that make it hard/easy? |
| **Value Proposition: Virtual vs F2F Care** | Patients: Compared to in-person visits, when would you prefer a video visit? Phone visit?  Providers: How do you determine for which clinical situations virtual care is preferential to in-person care? What about in-person care versus virtual? What outcomes signal appropriate use? |
| **Adopter System** | Patients: How hard is it for you to get care by video visits?  Providers: What changes in staff roles are implied when providing video visit care to women? |
| **Organizational  Capacity** | Provider: For women, what are the barriers and facilitators to virtual care? What changes will be needed for team interactions and routines to support regular virtual care use for women? |
| **Primacy of racialization** | Patients and Providers: How do racism-related exposures of bias and discrimination contribute to suboptimal uptake of virtual care among women? What structures support these biases? How does this impact women’s preferences and access to virtual care delivered in VA? |
| **Ordinariness of Racism** | Patients: How have you experienced discrimination or bias in receiving your healthcare from VA? What was that like? How did this impact your desire to access video or phone-delivered care? |
| **Wider Context** | Patient and Provider: What is the some of the larger forces (e.g., benefits, regulations, social drivers of health) at play outside the VA that influence the optimal use of virtual care for women? |
| **Adaptation** | Providers: How much opportunity do you feel you or your team has for adapting the use of virtual care for women over time? |
| **Intersectionality and**  **Sociodemographic**  **Characteristics** | Patients: What is your age? What is your race/ethnicity? What is the highest level of education you have obtained? What is your current employment status? How many members are in your household? In what branch of the military did you serve, and during what era? What is your gender identity? What are the other underrepresented groups you identify with?  Providers: What is your race/ethnicity? What is your field/discipline? How many years have you been in practice? How many years have you worked at VA? What are the other underrepresented groups you identify with?  Patient and Provider: How has your multiple social identities (e.g., gender, race) impacted your use of virtual care? |
| **Comfort with Technology** | Patients and Providers: How comfortable are you with the technology required for telehealth visits? What are some of the things that make harder or easier for you to use technology of telehealth visits? |
| **Adapted from Greenhalgh et al. J Med Internet Res 2017;19(11):e367* | |

Preliminary Interview Guide

This is an interview guide for semi-structured qualitative interviews of participants as laid out in the study protocol. The interviewer will use this guide to organize the interview, but exact questions may vary during the course of the interview or order may vary somewhat depending on answers given by participants. The interviewer will use the guide to make sure all essential domains are address, yet, will also follow the conversation, which may stray from the guide as the interviewer feels is appropriate.
